# *Review*: Pancreatic β-Cell Neogenesis Revisited

**DOI:** 10.1080/15438600490455079

**Published:** 2004

**Authors:** Maryline Paris, Cécile Tourrel-Cuzin, Cédric Plachot, Alain Ktorza

**Affiliations:** 1 Laboratoire de Physiopathologie de la Nutrition CNRS-UMR 7059 Universite Paris 7 2, place Jussieu, Tour 23-33, 1er etage, case 7126 Paris 75251 France

## Abstract

*β*-cell neogenesis triggers the generation of new *β*-cells from precursor cells. Neogenesis from duct epithelium is the most currently described and the best documented process of differentiation of precursor cells into *β*-cells. It is contributes not only to *β*-cell mass expansion during fetal and nonatal life but it is also involved in the maintenance of the *β*-cell mass in adults. It is also required for the increase in *β*-cell mass in situations of increase insulin demand (obesity, pregnancy). A large number of factors controlling the differentiation of *β*-cells has been identified. They are classified into the following main categories: growth factors, cytokine and inflamatory factors, and hormones such as PTHrP and GLP-1. The fact that intestinal incretin hormone GLP-1 exerts a major trophic role on pancreatic *β*-cells provides insights into the possibility to pharmacologically stimulate *β*-cell neogenesis. This could have important implications for the of treatment of type 1 and type 2 diabetes. Transdifferentiation, that is, the differentiation of already differentiated cells into *β*-cells, remains controversial.However, more and more studies support this concept. The cells, which can potentially “transdifferentiate” into *β*-cells, can belong to the pancreas (acinar cells) and even islets, or originate from extra-pancreatic tissues such as the liver. Neogenesis from intra-islet precursors also have been proposed and subpopulations of cell precursors inside islets have been described by some authors. Nestin positive cells, which have been considered as the main candidates, appear rather as progenitors of endothelial cells rather than *β*-cells and contribute to angiogenesis rather than neogenesis. To take advantage of the different differentiation processes may be a direction for future cellular therapies. Ultimately, a better understanding of the molecular mechanisms involved in *β*-cell neogenesis will allow us to use any type of differentiated and/or undifferentiated cells as a source of potential cell precursors.


*Experimental Diab. Res.*, 5:111–121, 2004
Copyright © Taylor and Francis Inc.
ISSN: 1543-8600 print / 1543-8619 online
DOI: 10.1080/15438600490455079

### *Review*: Pancreatic $\beta$-Cell Neogenesis Revisited

**Maryline Paris, Cécile Tourrel-Cuzin, Cédric Plachot, and Alain Ktorza**

*Laboratoire de Physiopathologie de la Nutrition, Universite Paris 7, Paris, France*

**$\beta$-cell neogenesis triggers the generation of new $\beta$-cells from precursor cells. Neogenesis from duct epithelium is the most currently described and the best documented process of differentiation of precursor cells into $\beta$-cells. It is contributes not only to $\beta$-cell mass expansion during fetal and nonatal life but it is also involved in the maintenance of the $\beta$-cell mass in adults. It is also required for the increase in $\beta$-cell mass in situations of increase insulin demand (obesity, pregnancy). A large number of factors controlling the differentiation of $\beta$-cells has been identified. They are classified into the following main categories: growth factors, cytokine and inflamatory factors, and hormones such as PTHrP and GLP-1. The fact that intestinal incretin hormone GLP-1 exerts a major trophic role on pancreatic $\beta$-cells provides insights into the possibility to pharmacologically stimulate $\beta$-cell neogenesis. This could have important implications for the of treatment of type 1 and type 2 diabetes. Transdifferentiation, that is, the differentiation of already differentiated cells into $\beta$-cells, remains controversial. However, more and more studies support this concept. The cells, which can potentially "transdifferentiate" into $\beta$-cells, can belong to the pancreas (acinar cells) and even islets, or originate from extra-pancreatic tissues such as the liver. Neogenesis from intra-islet precursors also have been proposed and subpopulations of cell precursors inside islets have been described by some authors. Nestin positive cells, which have been considered as the main candidates, appear rather as progenitors of endothelial cells rather than $\beta$-cells and contribute to angiogenesis rather than neogenesis. To take advantage of the different differentiation processes may be a direction for future cellular therapies. Ultimately, a better understanding of the molecular mechanisms involved in $\beta$-cell neogenesis will allow us to use any type of differentiated and/or undifferentiated cells as a source of potential cell precursors.**

**Keywords** Ductal Cell Precursors; GLP-1; Intra-Islet Precursors; Nestin; Transdifferentiation

$\beta$-cell neogenesis is the mechanism that triggers the generation of new $\beta$-cells from precursor cells. Although this definition is largely accepted, the origin and the nature of the cell precursors are controversial. For a long time, $\beta$-cell neogenesis was considered as a process restricted to the mechanism allowing morphological and functional changes of ductal epithelial undifferentiated precursors into $\beta$-cell [1, 2]. However, recent data and new concepts suggest that $\beta$-cell precursors could not be located only within pancreatic ducts [3, 4].

Taken into account the most recent findings and hypotheses, new $\beta$-cells could potentially originate from:

1. Ductal cells by ductal neogenesis.
2. Already differentiated pancreatic cell (i.e., exocrine, acinar or ductal) or extra-pancreatic cells through a mechanism so called "transdifferentiation."
3. An islet precursor cell by intra-islet neogenesis.

Figure 1 summarizes these different possibilities.

In this review we will focus on the mechanism and on the different potential precursors involved in $\beta$-cell neogenesis. We will also discuss the extent to which a better understanding

List of abbreviations: CK: Cytokeratin; c-kitR: c-kit receptor; EGF: Epidermal Growth Factor; EGFR: Epidermal Growth Factor Receptor; GLP-1: Glucagon like-peptide 1; GK rats: Goto-Kakisaki rats; HGF: Hepatocyte Growth Factors; INGAP: Islet Neogenesis-associated protein; INF-$\gamma$: Interferon-gamma; KGF: Keratinocyte Growth Factor; NGF: Nerve Growth Factor; NOD mice: Non-obese Diabetic mice; PDX-1: Pancreatic Duodenal Homeobox-1 (or IDX-1: Islet Duodenum Homeobox-1); PTHrP: Parathyroid hormone-related protein; Reg: Regenerating Factor; STZ: Streptozotocin; TGF-$\alpha$: Transforming Growth Factor-alpha; TGF-$\beta$: Transforming Growth Factor-beta; TNF-$\alpha$: Tumor Necrosis Factor-alpha; Trk-A: Tyrosine Kinase Receptor A.

Address correspondence to Alain Ktorza, Laboratoire de Physiopathologie de la Nutrition, CNRS-UMR 7059, Universite Paris 7, 2, place Jussieu, Tour 23-33, 1er etage, case 7126, 75251 Paris Cedex 05, France. E-mail: ktorza@paris7.jussieu.fr

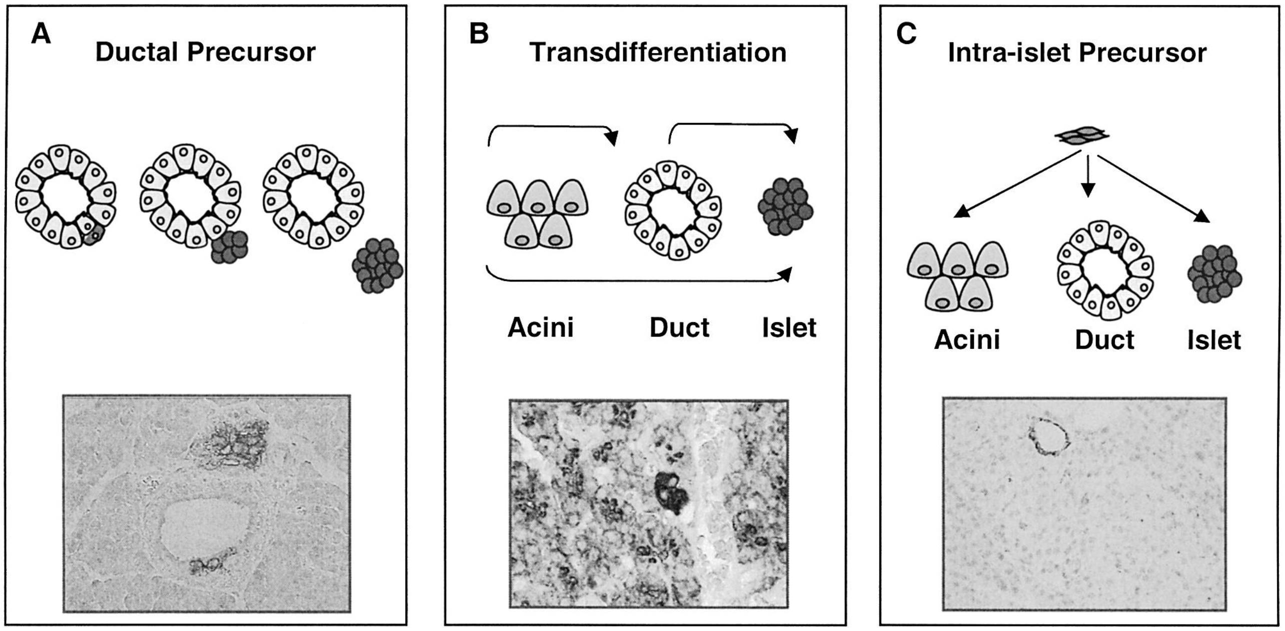

**FIGURE 1**
Different potential precursors of endocrine $\beta$-cells. (*A*) Ductal precursors are budding from the duct épithélium and express the differentiation maker Glut2. (*B*) Exocrine cells (amylase in gray) "transdifferentiate" into mature $\beta$-cells (insulin in dark gray) (*C*) Nestin, a putative intra-islet precursor of insulin-secreting cells (see text for the controversy).

of the mechanisms of $\beta$-cell neogenesis could help us in the possible outcomes for future treatments of diabetes based upon cell therapy.

### NEOGENESIS FROM DUCT EPITHELIUM

Neogenesis from duct epithelium is the most currently described and the best documented process by which progenitor cells can differentiate into endocrine cells.

### General Mechanism

*Fetus*

During fetal life [5], and in the neonatal period [6], intense $\beta$-cell differentiation is the major contributor to $\beta$-cell mass expansion. Two mechanisms are proposed. The first one involves the emergence of cells budding from the duct epithelium, and expressing islet hormones, especially insulin [2, 7]. These duct cells able to differentiate into $\beta$-cells are called "precursor cells." According to a second mechanism, that has been described only during the fetal life, the source of $\beta$-cells comes from a pool of proliferating cells expressing cytokeratin (CK) and located near the ductal tree [8]. Although it is difficult to assess what is the respective contribution of each of these mechanisms in the expansion of $\beta$-cell during the fetal stage, it is admitted that the first mechanism is the most current.

*Adult*

It is now well documented that neogenesis from ducts is involved in the maintenance of the $\beta$-cell mass and contributes to its expansion in situations of increased insulin demand in adult mammals including humans (reviewed in 9). Besides evident limitations in the use of human tissue samples, the regulation of $\beta$-cell differentiation in the adult stage is now better understood, thanks to the use of animal models of pancreatic regeneration and adaptation which stressed that $\beta$-cell differentiation from ductal cells can be strongly reactivated. Most importantly, using specific models it is possible to recapitulate in the adult situation, sequences found to occur during the development of the endocrine pancreas i.e., a wave of ductal proliferation followed by a subsequent differentiation of hyperplastic duct cells. The main models are: transgenic mouse over-expressing INF$\gamma$ [10], TGF$\alpha$ and gastrin over-expression [11], IL-6 or TNF$\alpha$ over-expression [12, 13], partial pancreatectomy [14], cellophane wrapping [15], main duct ligation [16, 17], and chronic glucose infusion in adult rats [18–20], which is a model of pancreatic adaptation without initial injury of the pancreas. Using the latest model, it was shown that 24 h of glucose infusion were enough to double the $\beta$-cell mass only by a stimulation of the neogenic process, $\beta$-cell proliferation being of minor importance. It was also demonstrated that hyperinsulinemia and hyperglycemia alone or in combination are able to dramatically activate neogenesis and then increase the $\beta$-cell mass, probably through specific ways [20].

Although the different situations of pancreas remodeling greatly contributed to the understanding of how $\beta$-cells differentiate in the adult situation, the question regarding the presence and the location of true pancreatic stem cells in the adult remains unanswered or, at least, largely controversial. For some authors there is a pool of resting precursor cells inside ducts, which has the ability to differentiate into $\beta$-cell upon a specific stimulus [1, 3]. For other authors, all the ductal cells are potentially precursors and are able to differentiate into $\beta$-cells [21, 22]. These cells are maintained in a quiescent status by presence of TGF$\beta$, but proliferation and differentiation of these cells can be reactivated upon the action of specific regulating factors as described in Figure 2 [23]. Interesting recent observations by Noguchi et al. [24] ascribe a particular role to the islet master gene PDX-1 in $\beta$-cell neogenesis from ductal cells. Indeed, the authors showed that PDX-1 protein possesses a protein transduction sequence in its structure, which allows it to permeate cells. They also showed that transduced PDX-1 into culture of pancreatic ducts triggered insulin gene expression. This suggests that ductal PDX-1 expression can have paracrine effects on (potentially progenitor?) neighboring cells within the pancreatic duct and then may induce the first steps of $\beta$-cell differentiation. This is an attractive hypothesis, which needs however, further confirmation.

Currently there is no unique reliable method to directly quantify the ductal-to $\beta$-cell neogenesis. This is due to the complexity of the mechanism, the involvement of many regulating factors, and many cell intermediates, that are not universal but may change according to the situation. The best way to estimate the activation of $\beta$-cell neogenesis from ducts is to evaluate concomitantly key parameters such as: proliferation rate of ductal cells, number of isolated $\beta$-cells or $\beta$-cell clusters budding from ducts [25], number of ductal cells co-expressing several pancreatic hormones [26], and finally the expression in the duct cells of transcription factors involved in the differentiation of $\beta$-cells.

### Ductal Cell Precursors

The characterization of the ductal precursors has become one of the major challenges for the upcoming research in $\beta$-cell differentiation. The phenotype of these precursors is difficult to identify and it seems to be different between fetus and adult. In the fetus, markers for these precursors are Glut-2, TrkA, and vimentin when co-expressed with CK20 [27]. In the adult no real marker for ductal cell precursors has been identified. In fact a co-expression of CK20-Insulin or CK20-Glucagon has been described in ductal cells, however these cells were already in a late stage of differentiation, making this co-expression not an early event of ductal-to $\beta$-cell neogenesis. In any case, the adult cell precursor and especially the ductal cell precursors are very difficult to identify probably because of the heterogeneous cell population that expressed different markers at the different stage of the $\beta$-cell differentiation [28].

### Factors Regulating Neogenesis from Duct Epithelium

A large number of factors controlling the differentiation of $\beta$-cells has been identified. Currently, these factors are classified in the following main categories: growth factors (i.e., TGF$\beta$, TGF$\alpha$, EGF, HGF, NGF, IGFs, and VEGF), regeneration factors (i.e., INGAP, Reg), cytokine and inflammatory factors (i.e., TNF$\alpha$, IL-6, INF$\gamma$), and other hormones such as PTHrP and GLP-1. It is obviously impossible to recapitulate all these factors and their possible actions. Therefore, we decided to concentrate here on GLP-1 because of the increasing interest for this factor as a stimulator of $\beta$-cell growth and its potential use as a therapeutic agent in type 2 diabetes (detailed information and references in Table 1).

### *GLP-1 and $\beta$-Cell Growth*

GLP-1 is an intestinal incretin hormone derived from the processing of proglucagon, that exerts insulinotropic actions on insulin-producing pancreatic islet $\beta$-cells (reviewed in 29). The importance of GLP-1 for stimulation of islet cell proliferation was originally demonstrated in lean 20-day old normoglycemic mice [30]. Afterwards, several studies using in vivo models showed that GLP-1 can regulate islet growth mainly by controlling $\beta$-cell neogenesis [31–35]. Our own observations, using a recognized model of $\beta$-cells regeneration (neonatal Wistar rats injected with streptozotocin, so-called n0-STZ), have shown that GLP-1 and Exendin-4, applied during the neonatal period, strongly stimulate $\beta$-cell regeneration mainly by $\beta$-cell neogenesis [35]. Furthermore, treatment of diabetic Goto-Kakizaki (GK) rats with GLP-1 or Exendin-4 from day 2 to day 6 after birth, resulted in stimulation of $\beta$-cell neogenesis and proliferation with persistent expansion of $\beta$-cell mass detected at adult age [34]. Altogether these suggest that GLP-1 and its analogs exert a major trophic role on pancreatic $\beta$-cells, in addition to theirs well-known effects on insulin biosynthesis and release. The use of GLP-1 analogs gives for the first time the possibility to pharmacologically stimulate $\beta$-cells neogenesis from precursor cells, even in diabetic adults.

### *GLP-1 and $\beta$-Cell Differentiation from Cell Lines*

Several studies have shown that incubation of pancreatic exocrine cell lines with GLP-1 or Exendin-4 promotes differentiation of these cells to an endocrine phenotype. AR42J cells (rat pancreatic acinar cell line) treatment with GLP-1 or

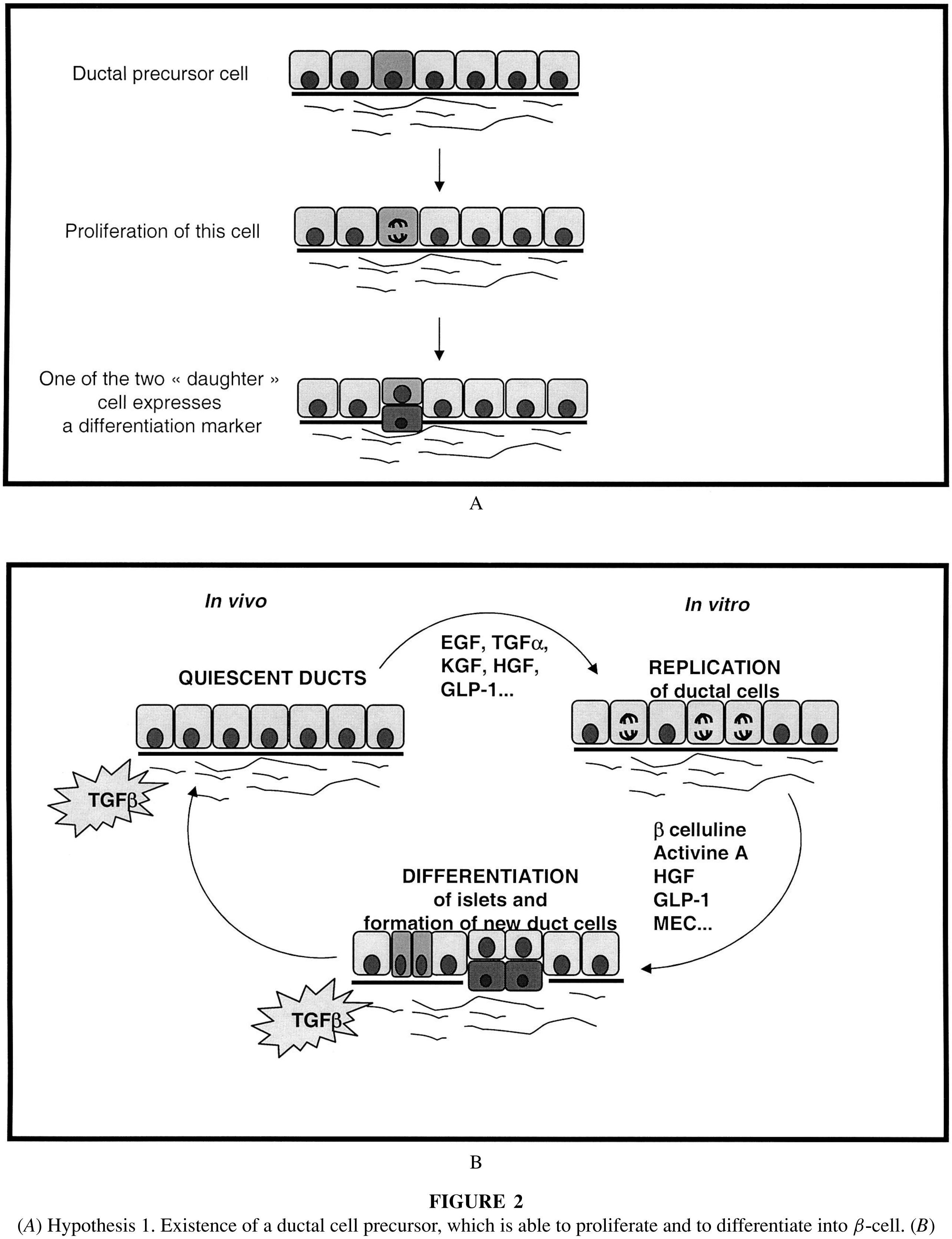

**FIGURE 2**
(*A*) Hypothesis 1. Existence of a ductal cell precursor, which is able to proliferate and to differentiate into $\beta$-cell. (*B*) Hypothesis 2. Quiescent ductal cells are able to prolifération and differentiate into $\beta$-cells under the action of several different factors (adapted from reference 23).

**TABLE 1**
Trophic role of GLP-1 and analogs on pancreatic $\beta$-cells. Main animal models and references

| Animal models used | Actions of GLP-1 or analogs | References |
|---|---|---|
| Umea ob/+ or +/+ | Increase of the pancreatic islet growth | Edvell et al., 1999 |
| Partially pancreatectomized rat (90% Px rats) | Stimulation of $\beta$-cell replication and neogenesis | Xu et al., 1999 |
| db/db mouse, GLP-1 receptor null mouse | Stimulation of PDX-1 expression while stimulating neogenesis | Stoffers et al., 2000 |
| Aging Wistar rats | Increased of pancreatic $\beta$-cell mass by stimulating $\beta$-cell differentiation just at the back of $\beta$-cell | Perfetti et al., 2000 |
| Neonatal Wistar rat injected with streptozotocin (n0-STZ) | Short- and long-term beneficial effects on $\beta$-cell mass recovery | Tourrel et al., 2001 |
| Goto-Kakizaki rat (GK rat) | Delay of the onset of type 2 diabetes by expansion of $\beta$-cell mass | Tourrel et al., 2002 |

Exendin-4 induces their differentiation into islet-like cells. Differentiated cells exhibit increased expression of $\beta$-cell genes and the capacity to release insulin [36]. Similar experiments were carried out using two pancreatic ductal cell lines, rat ARIP and human PANC-1 cells [37]. Interestingly, the differentiation of PANC-1 cells which are PDX-1 negative into pancreatic $\beta$-like cells after GLP-1 or Exendin-4 treatment required transfection with human IDX-1 (PDX-1), whereas ARIP cells, which are naturally PDX-1 positive, spontaneously differentiated into insulin-secreting cells after GLP-1 exposure. Finally, in Capan-1 cells derived from human pancreatic ductal carcinoma, exposed to Exendin-4 during several days, the number of cells containing insulin and glucagon increased to 10% from 40% in the basal state [38].

*GLP-1 and $\beta$-Cell Differentiation from Fetal and Adult Islet Precursors*

Several studies used fetal islet cell precursors to examine whether exposure to GLP-1 or analogs is associated with enhanced differentiation of previously immature islet precursors. Thus, Exendin-4 has been shown to enhance PDX-1 expression in human islet-like cell clusters treated for 4 days in vitro. After transplantation of these cell clusters under the kidney capsule of athymic rats, a 10-day treatment with exendin-4 induced functional maturation of transplanted cells and growth of clusters, as assessed 8 weeks following the transplant [39]. Other experiments showed that the treatment with GLP-1 can induce differentiation and maturation of fetal pig islet clusters during culture. Furthermore, transplantation of these in vitro treated cells into immunodeficient mice revealed an enhanced insulin secretion when exposed to glucose in vivo [40]. Recently, Abraham et al. reported the expression of functional GLP-1 receptor into nestin positive islet-derived progenitor cells (NIPs) and that GLP-1 stimulate the differentiation of NIPs into insulin-producing cells [41].

In summary, a large amount of evidence suggest that administration of GLP-1 or agonist promotes differentiation of functional of $\beta$-cells in vitro and vivo, thus strengthening the possibility that GLP-1 and analogs may be useful for production of new $\beta$-cells and has important implications for the treatment of type 1 and type 2 diabetes.

### TRANSDIFFERENTIATION

### Definition

Transdifferentiation (Figure 3) was first defined as the transforming process from a worm stage into an insect. Now the use of this termination has been enlarged, and applied for lot of organs in different species. One of the best examples is the transdifferentiation from retinal cell epithelium into neuron in the chicken [42]. The relevance of this process for the generation of new $\beta$-cells remains somewhat controversial but recent studies, that will be reported below, support this concept. According to these studies, the cells which can potentially "transdifferentiate" into $\beta$-cells can belong to the pancreas, and even the islet, itself or originate from extra-pancreatic tissues such as the liver.

### Intra-Pancreatic Transdifferentiation

*Endocrine Cells into Duct Cells*

Using a tri-dimensional cell culture system, Yuan et al. have observed that isolated islet from post mortem patients were able to transdifferentiate into ductal cells [43]. These ductal cells

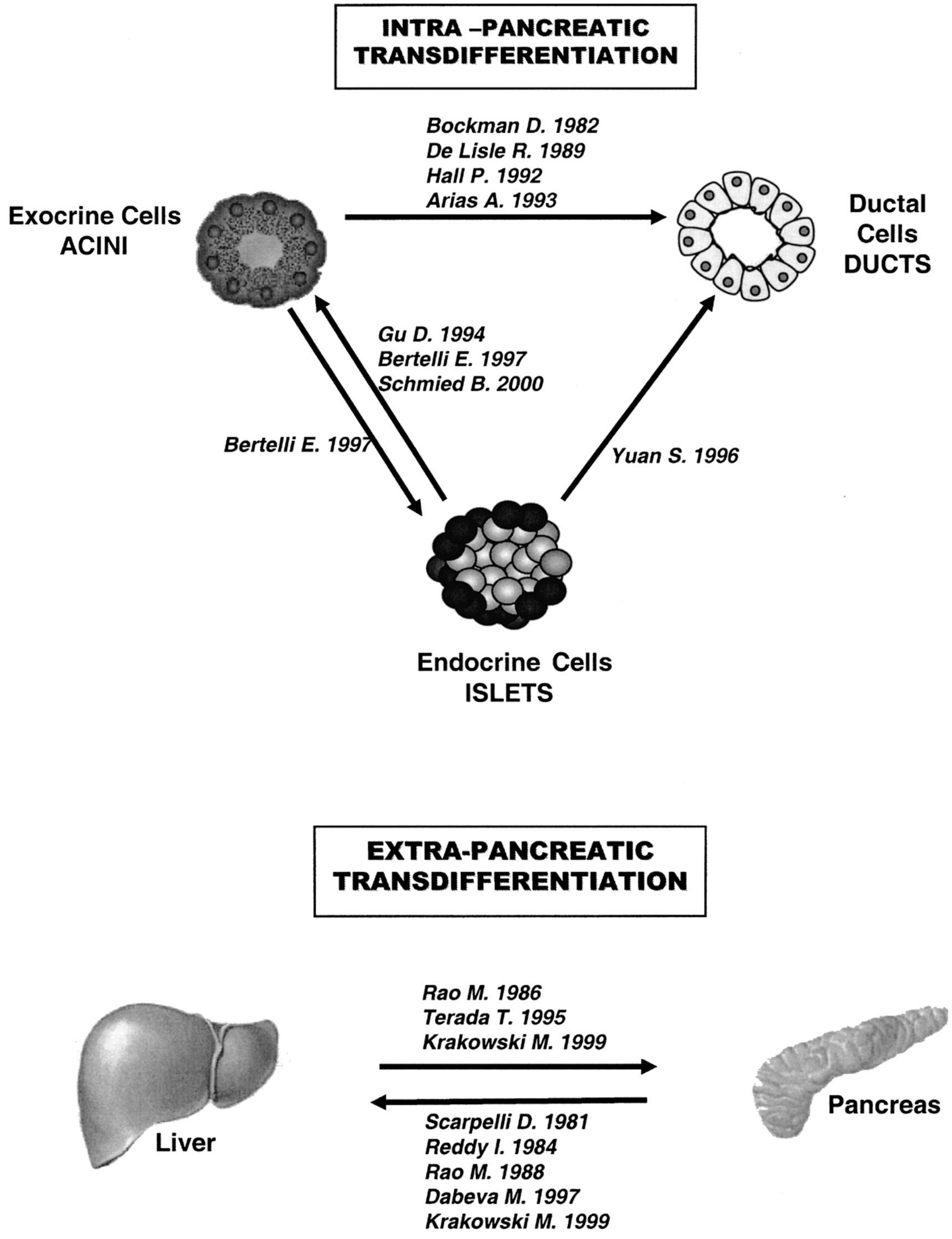

**FIGURE 3**

Schematic representation of the different possibilites of intra and extra pancreatic transdifferentiation in vivo.

presented particular properties since they still expressed enolase which is a marker of the endocrine tissue. Moreover the authors observed the appearance of the CK19 a marker of the fetal ductal cell, suggesting that these cells were not fully mature but rather corresponded to cells expressing an intermediate phenotype.

*Endocrine Cells into Acinar Cells*

Using the pancreatic duct ligation model, Bertilli et al. have shown by electron microscopy and dual immunostaining the presence of cells co-expressing insulin and amylase in both endocrine and exocrine compartments [25]. Same results were observed in transgenic mice overexpressing INF$\gamma$ [44]. The presence of cells displaying both exocrine and endocrine markers emphasizes the existence of an intermediate step during the transdifferentiation process as found in experimental models of pancreas remodeling that are associated with a strong $\beta$-cell neogenesis activity (reviewed in 3). Finally, human islets maintained in vitro for a period of one year, could transdifferentiate in some particular conditions. Endocrine cells can transdifferentiate into exocrine cells and subsequently into an undifferentiated phenotype characterized by the expression of enolase, vimentin, CK7 and CK19, TGF$\alpha$ and EGFR. These undifferentiated cells are considered by the authors as precursor cells [45].

*Acinar Cells into Duct Cells*

Studies on the transdifferentiation of acinar cells into duct cells were initially performed essentially for the understanding of the earliest events that happened during the cancer of the pancreas. The study of 7 pancreases from patients suffering from chronic pancreatisis showed a cell transformation from an acinar phenotype into a ductal phenotype, as assessed by the reduction of the number of zymogene granules and the increase of the size of the lumen of the future duct [46]. Similarly, data from in vitro studies found that human adult acinar cells could transdifferentiate into ductal cells when cultured on a collagen matrix [47]. Same results were observed by Hall et al. using cultured human acinar cells [48], while Arias et al. described the same phenomenon using other species such as rats and guinea pigs [49].

*The AR42J Cell Model*

AR42J is a cell line displaying acinar characteristics. These cells are well known for their capacity to transdifferentiate into different cell types when cultured in appropriate environment. In the presence of activin A alone, they can turn into endocrine phenotype expressing the pancreatic polypeptide, while in presence of activin A and $\beta$-cellulin, AR42J cells transdifferentiate into insulin secreting cells [50]. Finally, in the presence of GLP-1, AR42J cells can turn into insulin and glucagon producing cells [36].

### Hepatocyte/Pancreas Transdifferentiation

During embryonic development cells that have the ability to transdifferentiate generally come from adjacent region. The transdifferentiation from hepatic cells to pancreas cells is probably one of the best documented [51] (Figure 3).

In 1986 Rao et al. observed the presence of pancreatic-like tissue in the liver of rats when treated by polychlorinated biphenyls, a carcinogenic agent. These cells organized in acini as shown by location of the nucleus at the basal pole of the cells and the presence of zymogen granules expressing amylase and trypsinogen [52]. More recently, expression of pancreatic enzymes ($\alpha$-amylase, trypsinogen and lipase) was identified in human liver during both development and maturation processes. Using transgenic mice overexpressing INF$\gamma$ and treated with KGF, Krakowski et al. observed the appearance of hepatocytes and duct cells in pancreatic islets. Interestingly, these insular duct cells displayed a high proliferative activity [53].

*Hepatocyte Cell Precursors into Pancreatic Cells*

Some cells of the liver Hering duct have the ability to proliferate and to regenerate upon hepatic lesion. These cells also called oval cells express some epithelial markers as CK19 and hepatic markers as albumin. Moreover, these cells are characterized by the expression on their plasma membranes of antigen such as, c-kitR and Thy-1, which are also express in hematopoïetic stem cells. These oval cells are able to differentiate in vitro into epithelial biliary duct cells and into hepatocytes. They are not considered as early cell precursors but more likely as cell precursors dependent on regenerative processes [51]. The most important characteristic of these oval cells is that they are also found in pancreas in response to specific stimuli. For example, in all models of transdifferentiation from liver into pancreas, oval cells are found in pancreas and more precisely in the ductal tree or adjacent to it [52, 54–56].

Recently, Yang et al. showed that hepatic oval cells in culture could differentiate into insulin secreting cells with most of the characteristics of mature $\beta$-cells. These cells could reverse diabetes when injected into a diabetic rat [57, 58]. Moreover, using an approach based on flow cytometry Suzuki et al. were able to isolate hepatic stem cells that retain the capacity to differentiate into pancreatic cells [59].

### INTRA-ISLET PRECURSORS

Tsanadis et al. have shown inside human fetal pancreatic islets the presence of a cell type morphologically different from the commonly described endocrine and exocrine cell types [4]. These cells look as hepatocyte-like cells descending from the ductal cell precursors observed in rat and hamster. The authors conclude that these cells were a new population of cell precursors inside human fetal islets. Fernandes et al. have described using two animal models, (streptozotocin-injected and NOD mice), the presence of intra-islets cell precursors. These precursors co-expressing somatostatin and PDX-1 are able to differentiate into $\beta$-cells when the pancreas has undergone a lesion [60]. Recently Guz et al. have described two potential cell precursors located inside islets using a model of pancreatic regeneration [61]. The first precursor-type could express GLUT-2 as previously described by Pang et al. [62], while the second could co-express somatostatin and insulin. Taken together these studies strongly suggest the possibility of an activation of neogenesis directly from precursor cells located inside islets. This process could be considered as transdifferentiation of endocrine non $\beta$-cells into $\beta$-cells.

Since a few years special attention was paid to intra-islet nestin positive cells and for some authors nestin was considered as a potential marker of precursor cells within the islet. Nestin is an intermediate filament protein identified as a marker for a multipotent stem cell population in the central nervous system [63]. Furthermore, nestin-expressing cells were reported in pancreatic islets of adult mice [64], rats, and humans [65]. Clonigenicity and multipotency, including the capacity to form new islet cells, were reported for nestin-positive cells from adult [65] and fetal [66] pancreas. Knowing the role of nestin-positive cells in central nervous system, nestin appeared as the "ideal" pancreatic precursor cells marker. However, nestin has also been described as a marker for reactive stellate cells, or pericytes, and endothelial cells during active angiogenesis [67]. Other authors reported that nestin is expressed in mesenchymal but not epithelial cell precursors during development and is thus not directly involved in islet neogenesis [68, 69]. Therefore the role of nestin-positive cells as endocrine pancreatic precursors remains very controversial and the most recent data cast doubts as to whether intra-islet nestin positive cells could be considered as precursor cells in $\beta$-cell neogenesis [70–72]. However, the role of nestin positive cells in $\beta$-cell neogenesis cannot be totally excluded. Indeed, on the basis of an increasing amount of studies [67, 71, 73], it can be proposed that nestin positive cells indirectly may contribute to the generation of new endocrine cells by promoting angiogenesis which in turn will promote neogenesis by secreting trophic factors.

In conclusion, although different observations suggest the presence of subpopulations of cell precursors inside islets of Langerhans, it has been impossible so far to isolate precursor cells from endocrine or ductal origins and the existence of such cells must be considered with caution.

### CONCLUSION

It has been long recognized that during late fetal and early neonatal life, neogenesis is the main process which insures $\beta$-cell growth. There is now increasing evidence that the neogenic process is also largely involved in $\beta$-cell mass homeostasis in the adult. Moreover, neogenesis appears as crucial for pancreas plasticity, which is a unique property of the endocrine pancreas to adapt the $\beta$-cell mass to increased secretory demand without hyperglycemia. This has been demonstrated repeatedly under physiological (e.g., pregnancy, obesity) and pathological (e.g., growth hormone and cortisol excess) conditions [9]. In experimental models of increased insulin demand (prolonged glucose infusion) neogenesis is even the only process allowing $\beta$-cell growth [19, 20].

The classical definition of endocrine cell neogenesis involves the emergence of endocrine cells from undifferentiated progenitor cells located in pancreatic ducts, which migrate into the exocrine pancreas and proliferate to form the islets of Langerhans. We attempted here to review some of the recent studies in vivo and in vitro which prompt us to think that the generation of new $\beta$-cells may be not restricted to this process and rather be the result of different mechanisms and require different pancreatic and extra-pancreatic precursor cells. If pancreatic ducts are the main source of precursor cells, it is unlikely that there is a unique precursor and, for the moment, a specific marker of these precursor cells is still lacking. The possible ability of PDX-1 to induce insulin gene expression in pancreatic ducts by a paracrine effect is a very interesting hypothesis, which deserves further studies.

If the search of intra-islet precursors is somewhat disappointing for the moment, the possibility of an activation of neogenesis directly from precursor cells located inside islets is supported by some studies [60–62]. Especially, the conversion of endocrine non $\beta$-cells into $\beta$-cells [61; Paris et al., personal communication] is a new way to explore. In addition, although nestin positive cells are very probably not intra-islet precursors of $\beta$-cells, the studies on these cells opened a new research field, i.e., the relationship between $\beta$-cell neogenesis and angiogenesis [71].

Finally, to take advantage of the transdifferentiation process, especially the transdifferentiation from acinar cells, as a mechanism to obtain a source of differentiated $\beta$-cells may be a direction for future cellular therapies. Acinar tissue in pancreas represents more than 95% of the mass of the organ and may be considered as a potential source of $\beta$-cell.

Ultimately, a better understanding of the molecular mechanisms involved in $\beta$-cell neogenesis will allow us to use any type of differentiated [74] and/or undifferentiated cells as a source of potential cell precursors.

### REFERENCES

[1] Bonner-Weir, S. (1994) Regulation of pancreatic beta-cell mass in vivo. *Recent Prog. Horm. Res.*, **49,** 91–104.

[2] Pictet, R. L., Clark, W. R., Williams, R. H., and Rutter W. J. (1972) An ultrastructural analysis of the developing embryonic pancreas. *Dev. Biol.*, **29,** 436–467.

[3] Bouwens, L. (1999) [Neogenesis of beta cells and islet formation]. *Journ. Annu. Diabetol. Hotel Dieu*, 1–12.

[4] Tsanadis, G., Kotoulas, O., and Lollis, D. (1995) Hepatocyte-like cells in the pancreatic islets: Study of the human foetal pancreas and experimental modesl. *Histol. Histopathol.,* **10,** 1–10.

[5] Swenne, I., and Eriksson, U. (1982) Diabetes in pregnancy: Islet cell proliferation in the fetal rat pancreas. *Diabetologia,* **23,** 525–528.

[6] Bouwens, L., Wang, R. N., De Blay, E., Pipeleers, D. G., and Kloppel, G. (1994) Cytokeratins as markers of ductal cell differentiation and islet neogenesis in the neonatal rat pancreas. *Diabetes,* **43,** 1279–1283.

[7] Slack, J. M. (1995) Developmental biology of the pancreas. *Development,* **121,** 1569–1580.

[8] Bouwens, L., and De Blay, E. (1996) Islet morphogenesis and stem cell markers in rat pancreas. *J. Histochem. Cytochem.,* **44,** 947–951.

[9] Bernard-Kargar, C., and Ktorza, A. (2001). Endocrine pancreas plasticity under physiological and pathological conditions. *Diabetes,* **50(Suppl 1),** S30–S35.

[10] Gu, D., and Sarvetnick, N. (1993) Epithelial cell proliferation and islet neogenesis in IFN-g transgenic mice. *Development,* **118,** 33–46.

[11] Wang, T. C., Bonner-Weir, S., Oates, P. S., Chulak, M., Simon, B., Merlino, G. T., Schmidt, E. V., and Brand, S. J. (1993) Pancreatic gastrin stimulates islet differentiation of transforming growth factor alpha-induced ductular precursor cells. *J. Clin. Invest.,* **92,** 1349–1356.

[12] Campbell, I. L., Hobbs, M. V., Dockter, J., Oldstone, M. B., and Allison, J. (1994) Islet inflammation and hyperplasia induced by the pancreatic islet-specific overexpression of interleukin-6 in transgenic mice. *Am. J. Pathol.,* **145,** 157–166.

[13] Higuchi, Y., Herrera, P., Muniesa, P., Huarte, J., Belin, D., Ohashi, P., Aichele, P., Orci, L., Vassalli, J. D., and Vassalli, P. (1992) Expression of a tumor necrosis factor alpha transgene in murine pancreatic beta cells results in severe and permanent insulitis without evolution towards diabetes. *J. Exp. Med.,* **176,** 1719–1731.

[14] Bonner-Weir, S., Trent, D. F., and Weir, G. C. (1983) Partial pancreatectomy in the rat and subsequent defect in glucose-induced insulin release. *J. Clin. Invest.,* **71,** 1544–1553.

[15] Rosenberg, L., and Vinik, A. I. (1992) Trophic stimulation of the ductular-islet cell axis: A new approach to the treatment of diabetes. *Adv. Exp. Med. Biol.*, **321,** 95–104; discussion 105–109.

[16] Boquist, L., and Edstrom, C. (1970) Ultrastructure of pancreatic acinar and islet parenchyma in rats at various intervals after duct ligation. *Virchows Arch. A Pathol. Pathol. Anat.*, **349,** 69–79.

[17] Wang, R. N., Kloppel, G., and Bouwens, L. (1995) Duct- to islet-cell differentiation and islet growth in the pancreas of duct-ligated adult rats. *Diabetologia,* **38,** 1405–1411.

[18] Bonner-Weir, S., Deery, D., Leahy, J. L., and Weir, G. C. (1989) Compensatory growth of pancreatic beta-cells in adult rats after short-term glucose infusion. *Diabetes,* **38,** 49–53.

[19] Bernard, C., Berthault, M. F., Saulnier, C., Ktorza, A. (1999) Neogenesis vs. apoptosis as main components of pancreatic beta cell ass changes in glucose-infused normal and mildly diabetic adult rats. *FASEB J.,* **13,** 1195–1205.

[20] Paris, M., Bernard-Kargar, C., Berthault, M. F., Bouwens, L., Ktorza, A. (2003) Specific and combined effects of insulin and glucose on functional pancreatic beta-cell mass in vivo in adult rats. *Endocrinology,* **144**(6), 2717–2727.

[21] Teitelman, G., Lee, J., and Reis, D. J. (1987) Differentiation of prospective mouse pancreatic islet cells during development in vitro and during regeneration. *Dev. Biol.,* **120,** 425–433.

[22] Bisgaard, H. C., and Thorgeirsson, S. S. (1991) Evidence for a common cell of origin for primitive epithelial cells isolated from rat liver and pancreas. *J. Cell. Physiol.,* **147,** 333–343.

[23] Bonner-Weir, S. (2000) Life and death of the pancreatic beta cells. *Trends Endocrinol. Metab.,* **11,** 375–378.

[24] Noguchi, H., Kaneto, H., Weir, G. C., and Bonner-Weir, S. (2003) PDX-1 protein containing its own antennapedia-like protein transduction domain can transduce pancreatic ducts and islet cells. *Diabetes,* **52,** 1732–1737.

[25] Bertelli, E., and Bendayan, M. (1997) Intermediate endocrine-acinar pancreatic cells in duct ligation conditions. *Am. J. Physiol.,* **273,** C1641–C1649.

[26] Bouwens, L., and Pipeleers, D. G. (1998) Extra-insular beta cells associated with ductules are frequent in adult human pancreas. *Diabetologia,* **41,** 629–633.

[27] Bouwens, L., Braet, F., and Heimberg, H. (1995) Identification of rat pancreatic duct cells by their expression of cytokeratins 7, 19, and 20 in vivo and after isolation and culture. *J. Histochem. Cytochem.*, **43,** 245–253.

[28] Bouwens, L., and Kloppel, G. (1996) Islet cell neogenesis in the pancreas. *Virchows Arch.*, **427,** 553–560.

[29] Drucker, D. J. (2003) Glucagon-like peptides: Regulators of cell proliferation, differentiation, and apoptosis. *Mol. Endocrinol.,* **17,** 161–171.

[30] Edvell, A., and Lindstrom, P. (1999) Initiation of increased pancreatic islet growth in young normoglycemic mice (Umea +/?). *Endocrinology,* **140,** 778–783.

[31] Xu, G., Stoffers, D. A., Habener, J. F., and Bonner-Weir, S. (1999) Exendin-4 stimulates both beta-cell replication and neogenesis, resulting in increased beta-cell mass and improved glucose tolerance in diabetic rats. *Diabetes,* **48,** 2270–2276.

[32] Stoffers, D. A., Kieffer, T. J., Hussain, M. A., Drucker, D. J., Bonner-Weir, S., Habener, J. F., and Egan, J. M. (2000) Insulinotropic glucagon-like peptide 1 agonists stimulate expression, of homeodomain protein IDX-1 and increase islet size in mouse pancreas. *Diabetes,* **49,** 741–748.

[33] Perfetti, R., Zhou, J., Doyle, M. E., and Egan, J. M. (2000) Glucagon-like peptide-1 induces cell proliferation and pancreatic-duodenum homeobox-1 expression and increases endocrine cell mass in the pancreas of old, glucose-intolerant rats. *Endocrinology,* **141,** 4600–4605.

[34] Tourrel, C., Bailbe, D., Lacorne, M. Meile M. J., Kergoat, M. and Portha, B. (2002) Persistent improvement of type 2 diabetes in the Goto-Kakizaki rat model by expansion of the beta-cell mass during the prediabetic period with glucagon-like peptide-1 or exendin-4. *Diabetes,* **51,** 1443–1452.

[35] Tourrel, C., Bailbe, D., Meile, M. J., Kergoat, M., and Portha, B. (2001) Glucagon-like peptide-1 and exendin-4 stimulate beta-cell neogenesis in streptozotocin-treated newborn rats resulting in persistently improved glucose homeostasis at adult age. *Diabetes,* **50,** 1562–1570.

[36] Zhou, J., Wang, X., Pineyro, M. A., and Egan, J. M. (1999) Glucagon-like peptide 1 and exendin-4 convert, pancreatic AR42J cells into glucagon- and insulin-producing cells. *Diabetes,* **48,** 2358–2366.

[37] Hui, H., Wright, C., and Perfetti, R. (2001) Glucagon-like peptide 1 induces differentiation of islet duodenal homeobox-1-positive pancreatic ductal cells into insulin-secreting cells. *Diabetes,* **50,** 785–796.

[38] Zhou, J., Pineyro, M. A., Wang, X., Doyle, M. E., and Egan, J. M. (2002) Exendin-4 differentiation of a human pancreatic duct cell line into endocrine cells: Involvement of PDX-1 and HNF3beta transcription factors. *J. Cell. Physiol.,* **192,** 304–314.

[39] Movassat, J., Beattie, G. M., Lopez, A. D., and Hayek, A. (2002) Exendin 4 up-regulates expression of PDX 1 and hastens differentiation and maturation of human fetal pancreatic cells. *J. Clin. Endocrinol. Metab.,* **87,** 4775–4781.

[40] Hardikar, A. A., Wang, X. Y., Williams, L. J., Kwok, J., Wong, R. Yao, M., and Tuch, B. E. (2002) Functional maturation of fetal porcine beta-cells by glucagon-like peptide 1 and cholecystokinin. *Endocrinology,* **143,** 3505–3514.

[41] Abraham, E. J., Leech, C. A., Lin, J. C., Zulewski, H., and Habener, J. F. (2002) Insulinotropic hormone glucagon-like peptide-1 differentiation of human pancreatic islet-derived progenitor cells into insulin-producing cells. *Endocrinology,* **143,** 3152–3161.

[42] Eguchi, G., and Okada, T. S. (1973) Differentiation of lens tissue from the progeny of chick retinal pigment cells cultured in vitro: A demonstration of a switch of cell types in clonal cell culture. *Proc. Natl. Acad. Sci. U.S.A.,* **70,** 1495–1499.

[43] Yuan, S., Rosenberg, L., Paraskevas, S., Agapitos, D., and Duguid, W. P. (1996) Transdifferentiation of human islets to pancreatic ductal cells in collagen matrix culture. *Differentiation,* **61,** 67–75.

[44] Gu, D., Lee, M. S., Krahl, T., and Sarvetnick, N. (1994) Transitional cells in the regenerating pancreas. *Development,* **120,** 1873–1881.

[45] Schmied, B. M., Liu, G., Matsuzaki, H., Ulrich, A., Hernberg, S., Moyer, M. P., Weide, L., Murphy, L., Batra, S. K., and Pour, P. M. (2000) Differentiation of islet cells in long-term culture. *Pancreas,* **20,** 337–347.

[46] Bockman, D. E., Boydston, W. R., and Anderson, M. C. (1982) Origin of tubular complexes in human chronic pancreatitis. *Am. J. Surg*., **144,** 243–249.

[47] De Lisle, R. C., and Logsdon, C. D. (1990) Pancreatic acinar cells in culture: Expression of acinar and ductal antigens in a growth-related manner. *Eur. J. Cell. Biol.,* **51,** 64–75.

[48] Hall, P. A., and Lemoine, N. R. (1992) Rapid acinar to ductal transdifferentiation in cultured human exocrine pancreas. *J. Pathol.,* **166,** 97–103.

[49] Arias, A. E., and Bendayan, M. (1993) Differentiation of pancreatic acinar cells into duct-like cells in vitro. *Lab. Invest.,* **69,** 518–530.

[50] Mashima, H., Ohnishi, H., Wakabayashi, K., Mine, T., Miyagawa, J., Hanafusa, T., Seno, M., Yamada, H., and Kojima, I. (1996) Betacellulin and activin A coordinately convert amylase-secreting pancreatic AR42J cells into insulin-secreting cells. *J. Clin. Invest.,* **97,** 1647–1654.

[51] Grompe, M. (2003) Pancreatic-hepatic switches in vivo. *Mech. Dev.*, **120,** 99–106.

[52] Rao, M. S., Dwivedi, R. S., Subbarao, V., Usman, M. I, Scarpelli, D. G., Nemali, M. R., Yeldandi, A., Thangada, S., Kumar, S., and Reddy, J. K. (1988) Almost total conversion of pancreas to liver in the adult rat: A reliable model to study transdifferentiation. *Biochem. Biophys. Res. Commun.,* **156,** 131–136.

[53] Krakowski, M. L., Kritzik, M. R., Jones, E. M., Krahl, T., Lee, J., Arnush, M., Gu, D., and Sarvetnick, N. (1999) Pancreatic expression of keratinocyte growth factor leads to differentiation of islet hepatocytes and proliferation of duct cells. *Am. J. Pathol.,* **154,** 683–691.

[54] Dabeva, M. D., Hwang, S. G., Vasa, S. R., Hurston, E., Novikoff, P. M., Hixson, D. C., Gupta, S., and Shafritz, D. A. (1997) Differentiation of pancreatic epithelial progenitor cells into hepatocytes following transplantation into rat liver. *Proc. Natl. Acad. Sci. U.S.A.,* **94,** 7356–7361.

[55] Reddy, J. K., Rao, M. S., Qureshi, S. A., Reddy, M. K., Scarpelli, D. G., and Lalwani, N. D. (1984) Induction and origin of hepatocytes in rat pancreas. *J. Cell. Biol.,* **98,** 2082–2090.

[56] Scarpelli, D. G., and Rao, M. S. (1981) Differentiation of regenerating pancreatic cells into hepatocyte-like cells. *Proc. Natl. Acad. Sci. U.S.A.,* **78,** 2577–2581.

[57] Yin, L., Arenas, M., Jodoin, E., Zhou, J., Leffert, H., Sell, S., Peck, A., and Ramiya, V. (2001) The potentiality of liver oval cells to differentiate into insulin-producing cells. Abstract Book, *Conference on Pancreatic Development, Proliferation and Stem Cells, NIH, Bethesda, MD*, p. 48.

[58] Yang, L., Li, S., Hatch, H., Ahrens, K., Cornelius, J. G., Petersen, B. E. and Peck, A. B. (2002) in vitro trans-differentiation of adult hepatic stem cells into pancreatic endocrine hormone-producing cells. *Proc. Natl. Acad. Sci. U.S.A.,* **99,** 8078–8083.

[59] Suzuki, A., Zheng, Yw. W., Kaneko, S., Onodera, M., Fukao, K., Nakauchi, H., and Tanigichi, H. (2002) Clonal identification and characterization of self-renewing pluripotent stem cells in the developing liver. *J. Cell. Biol.,* **156,** 173–184.

[60] Fernandes, A., King, L. C., Guz, Y., Stein, R., Wright, C. V., and Teitelman, G. (1997) Differentiation of new insulin-producing cells is induced by injury in adult pancreatic islets. *Endocrinology,* **138,** 1750–1762.

[61] Guz, Y., Nasir, I., and Teitelman, G. (2001) Regeneration of pancreatic beta cells from intra-islet precursor cells in an experimental model of diabetes. *Endocrinology,* **142,** 4956–4968.

[62] Pang, K., Mukonoweshuro, C., and Wong, G. G. (1994) Beta cells arise from glucose transporter type 2 (Glut2)-expressing epithelial cells of the developing rat pancreas. *Proc. Natl. Acad. Sci. U.S.A.,* **91,** 9559–9563.

[63] Lendahl, U., Zimmerman, L. B., and McKay, R. D. (1990) CNS stem cells express a new class of intermediate filament protein. *Cell,* **60,** 585–595.

[64] Hunziker, E., and Stein, M. (2000) Nestin-expressing cells in the pancreatic islets of Langerhans. *Biochem. Biophys. Res. Commun.*, **271,** 116–119.

[65] Zulewski, H., Abraham, E. J., Gerlach, M. J., Daniel, P. B., Moritz, W., Muller, B., Vallejo, M., Thomas, M. K., and Habener, J. F. (2001) Multipotential nestin-positive stem cells isolated from adult pancreatic islets differentiate ex vivo into pancreatic endocrine, exocrine, and hepatic phenotypes. *Diabetes,* **50,** 521–533.

[66] Huang, H., and Tang, X. (2003) Phenotypic determination and characterization of nestin-positive precursors derived from human fetal pancreas. *Lab. Invest.,* **83**(4), 539–547.

[67] Lardon, J., Rooman, I., and Bouwens, L. (2002) Nestin expression in pancreatic stellate cells and angiogenic endothelial cells. *Histochem. Cell. Biol.*, **117,** 535–540.

[68] Selander, L., and Edlund, H. (2002) Nestin is expressed in mesenchymal and not epithelial cells of the developing mouse pancreas. *Mech. Dev.,* **113,** 189–192.

[69] Piper, K., Ball, S. G., Turnpenny, L. W., Brickwood, S., Wilson, D. L., and Hanley, N. A. (2002) Beta-cell differentiation during human development does not rely on nestin-positive precursors: Implications for stem cell-derived replacement therapy. *Diabetologia,* **45,** 1045–1047.

[70] Gao, R., Ustinov, J., Pulkkinen, M. A., Lundin, K., Korsgren, O., Otonkoski, T. (2003). Characterization of endocrine progenitor cells and critical factors for their differentiation in human adult pancreatic cell culture. *Diabetes,* **52,** 2007–2015.

[71] Treutelaar, M. K., Skidmore, J. M., Dias-Leme, C. L., Hara, M., Zhang, L., Simeone, D., Martin, D. M., and Burant, C. F. (2003) Nestin-lineage cells contribute to the microvasculature but not endocrine cells of the islet. *Diabetes,* **52(10),** 2503–2512.

[72] Delacour, A., Nepote, V., Trumpp, A., and Herrera, P. L. (2004) Nestin expression in pancreatic exocrine cell lineages. *Mech. Dev.,* **121,** 3–14.

[73] Klein, T., Ling, Z., Heimberg, H., Madsen, O. D., Heller, R. S., and Serup, P. (2003) Nestin is expressed in vascular endothelial cells in the adult human pancreas. *J. Histochem. Cytochem.,* **51(6),** 697–706.

[74] Tosh, D., and Slack, J. M. (2002) How cells change their phenotype. *Nat. Rev. Mol. Cell. Biol.*, **3,** 187–194.

